# Clinical Significance and Potential Role of *LSM4* Overexpression in Hepatocellular Carcinoma: An Integrated Analysis Based on Multiple Databases

**DOI:** 10.3389/fgene.2021.804916

**Published:** 2022-01-13

**Authors:** Liang Chen, Yun-hua Lin, Guo-qing Liu, Jing-en Huang, Wei Wei, Zhong-hua Yang, Yi-ming Hu, Jia-heng Xie, Hong-zhu Yu

**Affiliations:** ^1^ Department of General Surgery, Fuyang Hospital Affiliated to Anhui Medical Universitsy, Fuyang, China; ^2^ The First Clinical Medical College, Guangxi Medical University, Nanning, China; ^3^ Department of General Surgery, Hospital of Traditional Chinese Medicine, Baise, China; ^4^ College of Pharmacy, Jiangsu Ocean University, Lianyungang, China; ^5^ Department of Burn and Plastic Surgery, The First Affiliated Hospital of Nanjing Medical University, Nanjing, China

**Keywords:** HCC, LSM4, prognosis, diagnostic, gene set enrichment analysis, co-expressed gene

## Abstract

**Background:** Hepatocellular carcinoma (HCC) is a solid tumor with high recurrence rate and high mortality. It is crucial to discover available biomarkers to achieve early diagnosis and improve the prognosis. The effect of *LSM4* in HCC still remains unrevealed. Our study is dedicated to exploring the expression of *LSM4* in HCC, demonstrating its clinical significance and potential molecular mechanisms.

**Methods:** Clinical information and LSM4 expression values of HCC were obtained from Gene Expression Omnibus (GEO) and The Cancer Genome Atlas (TCGA) databases. Survival analysis and receiver operating characteristic (ROC) curve analysis were applied to evaluate the prognostic and diagnostic significance of *LSM4*. Calculating pooled standardized mean difference (SMD) and performing summary receiver operating characteristic (sROC) curve analysis to further determine its expression status and diagnostic significance. *LSM4*-related co-expressed genes (CEGs) were obtained and explored their clinical significance in HCC. *LSM4*-associated pathways were identified through Gene set enrichment analysis (GSEA).

**Results:** Up-regulated *LSM4* was detected in HCC tissues (SMD = 1.56, 95% CI: 1.29–1.84) and overexpressed *LSM4* had excellent distinguishing ability (AUC = 0.91, 95% CI: 0.88–0.93). *LSM4* was associated with clinical stage, tumor grade, and lymph node metastasis status (*p* < 0.05). Survival analysis showed that high *LSM4* expression was related to poor overall survival (OS) of HCC patients. Cox regression analysis suggested that high *LSM4* expression may be an independent risk factor for HCC. We obtained nine up-regulated CEGs of *LSM4* in HCC tissues, and six CEGs had good prognostic and diagnostic significance. GSEA analysis showed that up-regulated *LSM4* was closely related to the cell cycle, cell replication, focal adhesion, and several metabolism-associated pathways, including fatty acid metabolism.

**Conclusion:** Overexpressed *LSM4* may serve as a promising diagnostic and prognostic biomarker of HCC. Besides, *LSM4* may play a synergistic effect with CEGs in promoting the growth and metastasis of HCC cells *via* regulating crucial pathways such as cell cycle, focal adhesion, and metabolism-associated pathways.

## Introduction

As one of the most prevalent solid malignancies, hepatocellular carcinoma (HCC) is a major cause of cancer-related deaths worldwide (Nordenstedt et al., 2010). Numerous considerable improvements have been accomplished in drug therapies and non-drug therapies for HCC treatment over the past decades. However, HCC was still ranked fourth in mortality, causing over 780,000 deaths per year based on the World Health Organization’s statistics in 2018 (Bray et al., 2018). To date, alpha-fetoprotein (AFP) is one of the most common serum biomarkers used for diagnosing HCC (Liu Y et al., 2021). Despite this, due to insufficient sensitivity and specificity, AFP detection remains far from achieving satisfactory role for early HCC. (Wang and Wei, 2020). Numerous liver diseases, such as acute hepatitis and cirrhosis, can also cause elevating serum AFP levels (Xie et al., 2018). Therefore, it is vital to discover the novel effective biomarkers, which are of great significance for the early diagnosis and improvement of prognosis of HCC patients.

Smith-like (LSM) proteins are called the RNA binding protein family, with 13 members (LSM1 ∼ LSM14B). These members are closely related to tumorigenesis and metastasis of multiple tumor types, such as breast cancer (BRCA), mesothelioma and lung squamous cell carcinoma (LUSC) (Streicher et al., 2007; Watson et al., 2008; Li et al., 2020; Ta et al., 2021). Nevertheless, the biological functions of LSM gene family members in tumors are not fully understood. The small nuclear ribonucleoprotein Sm-like4 (*LSM4*) has been shown to be involved in the occurrence and malignant procession of BRCA, pancreatic cancer (PC), and ovarian cancer (OC) (Xue et al., 2018; Ta et al., 2021). For example, the expression of *LSM4* in BRCA was significantly increased and was closely related to lymph node metastasis and tumor cell proliferation (Yin et al., 2021). In OC, *LSM4* knockdown blocked cell migration and invasion of OC cells, and *LSM4* overexpression reversed the inhibitory effect of circ_0,025,033 knockdown on the above process (Hou and Zhang, 2021). However, *LSM4* has not been fully studied in HCC. The clinical application value and underlying molecular mechanism of *LSM4* in HCC are still unclear. Therefore, we used multiple available online databases (TGCA, GEO, UALCAN, etc.) to evaluate the clinical value of *LSM4* in HCC and explore its underlying mechanisms.

This study comprehensively analyzed the *LSM4* expression in HCC by calculating the pooled SMD based on public datasets. Use ROC and sROC analysis to evaluate the diagnostic significance. Cox regression analysis and survival analysis were carried out to determine the correlation of *LSM4* expression and the prognosis of HCC patients. GSEA analysis was used to identify *LSM4*-associated pathways.

## Materials and Methods

### Data Collection Through Online Databases

The HCC datasets used in this study were obtained from the TCGA (https://cancergenome.nih.gov/) and GEO databases (https://www.ncbi.nlm.nih.gov/geo/). The inclusion criteria we developed were as follows: 1) human origin, 2) HCC and non-HCC tissues were included, 3) the *LSM4* expression data was included in the datasets. In addition, the exclusion criteria were as follows: 1) the sample size was insufficient (n < 6), 2) did not contain the expression data of *LSM4*, 3) the data could not be processed under the current conditions. Available datasets were collected strictly in accordance with the established inclusion and exclusion criteria.

### *LSM4* Expression Status in HCC and Non-HCC Tissues

R software (version 3.6.3) was used to perform an independent sample *t*-test on the *LSM4* expression data included in our study to make the comparison on the *LSM4* expression status between the non-HCC tissues and HCC tissues. The R package ggplot2 was used for visualization, and the results were presented in the form of box plots. Subsequently, based on the included GEO datasets, STATA 14.0 software was used to calculate the standard mean deviation (SMD) to detect the *LSM4* expression level in HCC comprehensively. When datasets included had huge heterogeneity (I^2^ > 0.50), we selected the random-effects model for analysis. Publication bias was detected using Egger and Begg tests, and *p*-value ≥ 0.05 indicated no publication bias.

### Genetic Alteration Analysis of *LSM4*

The online database cBioPortal for Cancer Genomics database (https://www.cbioportal.org/) could be used to explore the genetic alteration information in numerous cancers and provide visualization of the analysis results (Tang et al., 2020). We used a hepatocellular carcinoma (TCGA, PanCancer Atlas) study containing 372 samples to explore the genetic alteration and mutation types of *LSM4* with this online tool.

### Relationship Between Clinicopathological Characteristics and *LSM4* Expression

In our current study, UALCAN (http://ualcan.path.uab.edu/) database was applied to explore the relationship between the clinicopathological characteristics and *LSM4* expression in HCC.

### Diagnostic Significance of *LSM4* in HCC

For determining the capability of *LSM4* in distinguishing between HCC tissues and non-HCC tissues, we carried out the receiver operating characteristic (ROC) curve analysis using the pROC package for analysis, and then the ROC curve was drawn with the application of the ggplot2 package (for visualization). In addition, the summary receiver operating characteristic (sROC) curve was also drawn by us to comprehensively determine the diagnostic value of *LSM4* and calculated the specificity and sensitivity of *LSM4* through STATA 14.0. Meanwhile, we also carried out Deeks’ test to further detect the publication bias of the study.

### Prognostic Significance of *LSM4* in HCC

The clinical data of HCC in the TCGA database was used to determine the relationship between the patient’s prognosis and *LSM4* expression. In order to evaluate the prognosis, HCC samples with survival time and survival status are collected. Incorporate *LSM4* expression data and corresponding clinical information were included in the univariate Cox regression analysis, and then incorporate the prognostic-related features selected in the above step into the multivariate Cox regression analysis to further assess the association between the patient’s prognosis and *LSM4* expression. Besides, using the TCGA dataset and the GEPIA database (http://gepia.cancer-pku.cn/), the Kaplan-Meier tests were applied to compare the difference in Disease-Free Survival (DFS) and overall survival (OS) of HCC patients with different *LSM4* expressions.

### Identification Co-expressed Genes of *LSM4* and Its Clinical Value

The cBioPortal and the GEPIA databases were used to obtain the co-expressed genes (CEGs) of *LSM4* in HCC. Significantly CEGs should meet the following two criteria: 1) correlation coefficient≥0.60, 2) *p*-value <0.05. Finally, the intersection genes of *LSM4* positively correlated CEGs obtained from the two databases were used for further analysis. Briefly, the expressions level of CEGs in HCC tissues and matched non-HCC tissues were also explored in our study. Furtherly, the survival analysis and ROC curve analysis were carried out to detect the clinical significance of CEGs in HCC.

### Gene Set Enrichment Analysis

To identify the signaling pathways related to *LSM4* expression in HCC, Gene Set Enrichment Analysis (GSEA) was utilized. When meeting the criteria of the false discovery rate (FDR) was less than 0.25, and the *p*-value was less than 0.05, the genes were considered significantly enriched in this pathway. Our current study used the clusterProfiler package and the enrichplot package on the R platform for analysis and visualization, respectively.

## Result

### Included Datasets and Information

We collected a total of 18 eligible datasets from GEO and TCGA databases. All specimens were liver tissues collected from HCC individuals and matched non-HCC individuals. Table 1 showed the basic information of included datasets. The mean (Mean) and standard deviation (SD) of *LSM4* expression value in the HCC tissues and the matched non-HCC tissues contained in included datasets were displayed.

**TABLE 1:** Characteristics of included studies.

Study	Author	Last update	Platform	Non-HCC tissues			HCC tissues		
				N0	Mean0	SD0	N1	Mean1	SD1
TCGA	NR	NR	NR	50	4.771	0.374	374	6.313	0.728
GSE101685	Sen-Yung H	2021	GPL570	8	7.212	0.196	24	8.426	0.817
GSE115018	Zhao G	2018	GPL20115	12	1.139	0.193	12	1.620	0.339
GSE121248	Hui KM	2019	GPL570	37	9.009	0.307	70	9.515	0.465
GSE19665	Nagae G	2019	GPL570	10	7.831	0.311	10	8.748	0.349
GSE29721	Bhattacharyya B	2019	GPL570	10	7.500	0.200	10	8.103	0.367
GSE33006	Huang Y	2019	GPL570	3	11.606	0.515	3	10.147	0.180
GSE39791	Kim J	2018	GPL10558	72	8.577	0.453	72	9.324	0.645
GSE41804	Hodo Y	2019	GPL570	20	10.107	0.428	20	10.881	0.830
GSE45436	Wang HW	2019	GPL570	39	7.435	0.371	95	8.638	0.604
GSE46408	Jeng Y	2019	GPL4133	6	11.407	0.396	6	12.652	0.568
GSE55092	Melis M	2019	GPL570	91	8.360	0.573	49	9.211	1.006
GSE57957	Mah W	2018	GPL10558	39	10.173	0.544	39	11.035	0.809
GSE60502	Kao KJ	2018	GPL96	18	9.148	0.379	18	10.028	0.447
GSE76427	Grinchuk OV	2018	GPL10558	52	10.131	0.332	115	10.712	0.590
GSE84402	Qin W	2019	GPL570	14	7.070	0.656	14	8.362	1.048
GSE87630	Woo HG	2018	GPL6947	30	9.959	0.434	64	11.291	0.625
GSE89377	Eun J	2021	GPL6947	13	9.518	0.365	40	10.272	0.593

### Up-Regulated *LSM4* Expression in HCC

**FIGURE 1:**
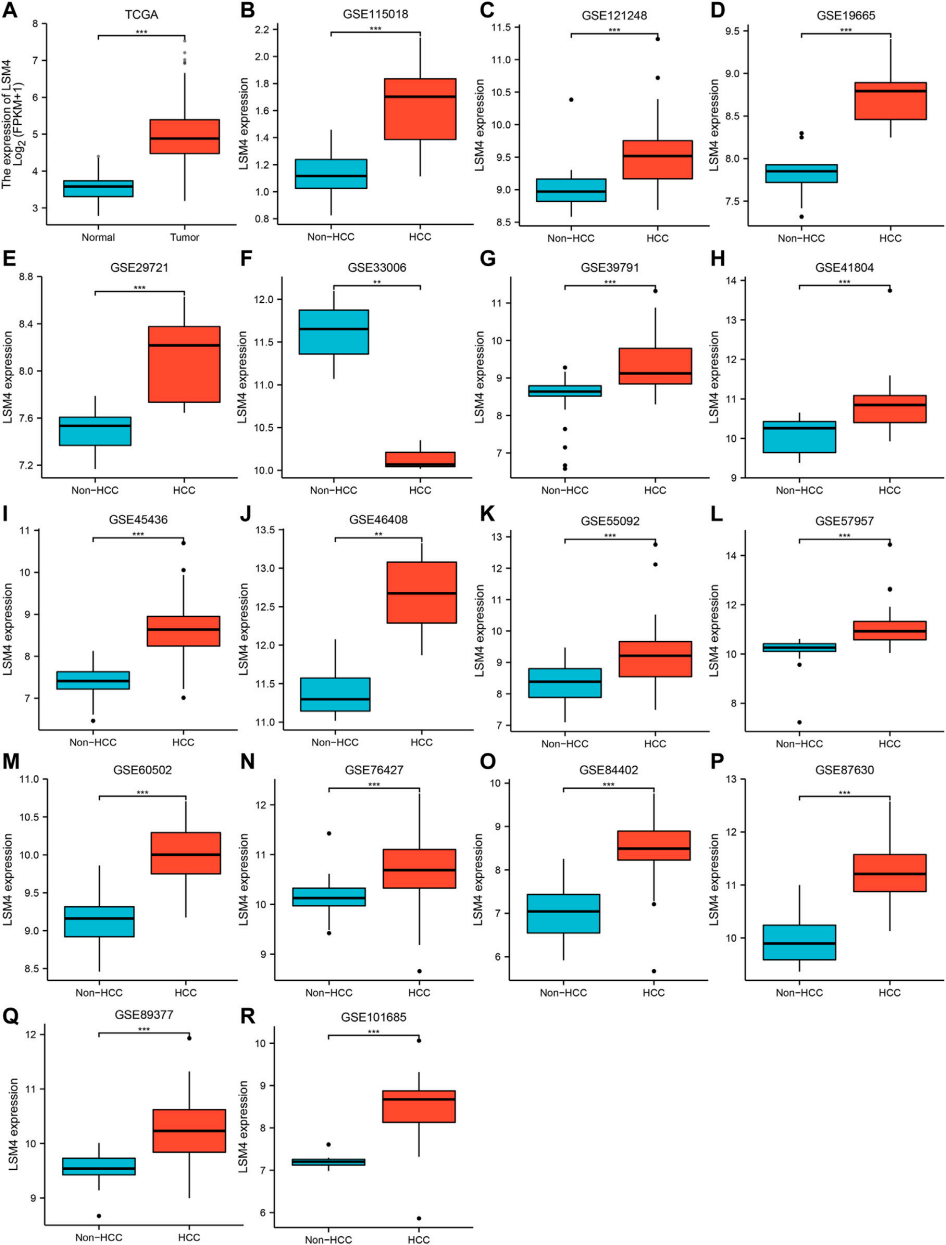
*LSM4* expression in hepatocellular carcinoma. *LSM4* expression between non-HCC tissues and HCC tissues based on GEO and TCGA datasets (*p < 0.05, **p < 0.01, ***p < 0.001). **(A)** TCGA. **(B)** GSE115018. **(C)** GSE121248. **(D)** GSE19665. **(E)** GSE29721. **(F)** GSE33006. **(G)** GSE39791. **(H)** GSE41804. **(I)** GSE45436. **(J)** GSE46408. **(K)** GSE55092. **(L)** GSE57957. **(M)** GSE60502. **(N)** GSE76427. **(O)** GSE84402. **(P)** GSE87630. **(Q)** GSE8937. **(R)** GSE101685.

**FIGURE 2:**
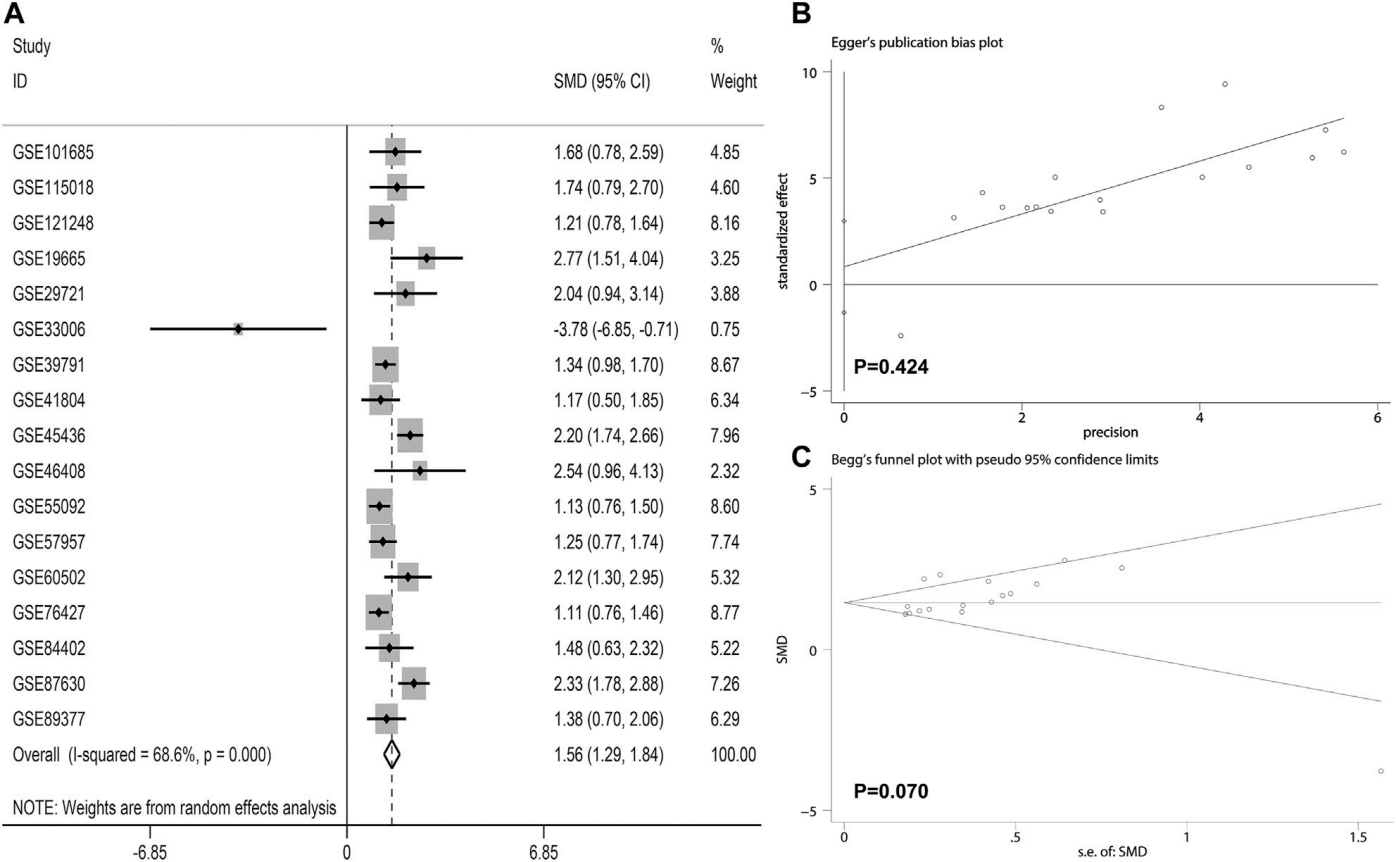
The integrated expression of *LSM4* evaluated through calculating pooled standardized mean difference (SMD) **(A)**; the Egger and Begg tests to evaluate publication bias **(B–C)**.

The *LSM4* expression levels in HCC tissues and non-HCC tissues were presented in Figure 1. Compared with non-HCC tissues, *LSM4* showed a significant up-regulation trend in 17 datasets (all *p*-value <0.05; Figure 1A–E, G–R). It was worth noting that the only dataset GSE33006 showed a significant down-regulation of *LSM4* expression (*p*-value <0.05; Figure 1F). The heterogeneity test results showed that I^2^ = 68.6% (*p*-value <0.0001), indicating significant heterogeneity. Therefore, based on 17 GEO datasets, random-effects analysis was applied to calculate the SMD. We observed that the HCC tissues had significantly higher *LSM4* expression levels than the matched non-HCC tissues (Figure 2A, SMD = 1.56, 95% CI: 1.29–1.84). The *p*-values of Begg and Egger tests were 0.070 and 0.424, respectively, indicating no publication bias (Figures 2B,C).

### Genetic Alteration Analysis of *LSM4*

**FIGURE 3:**
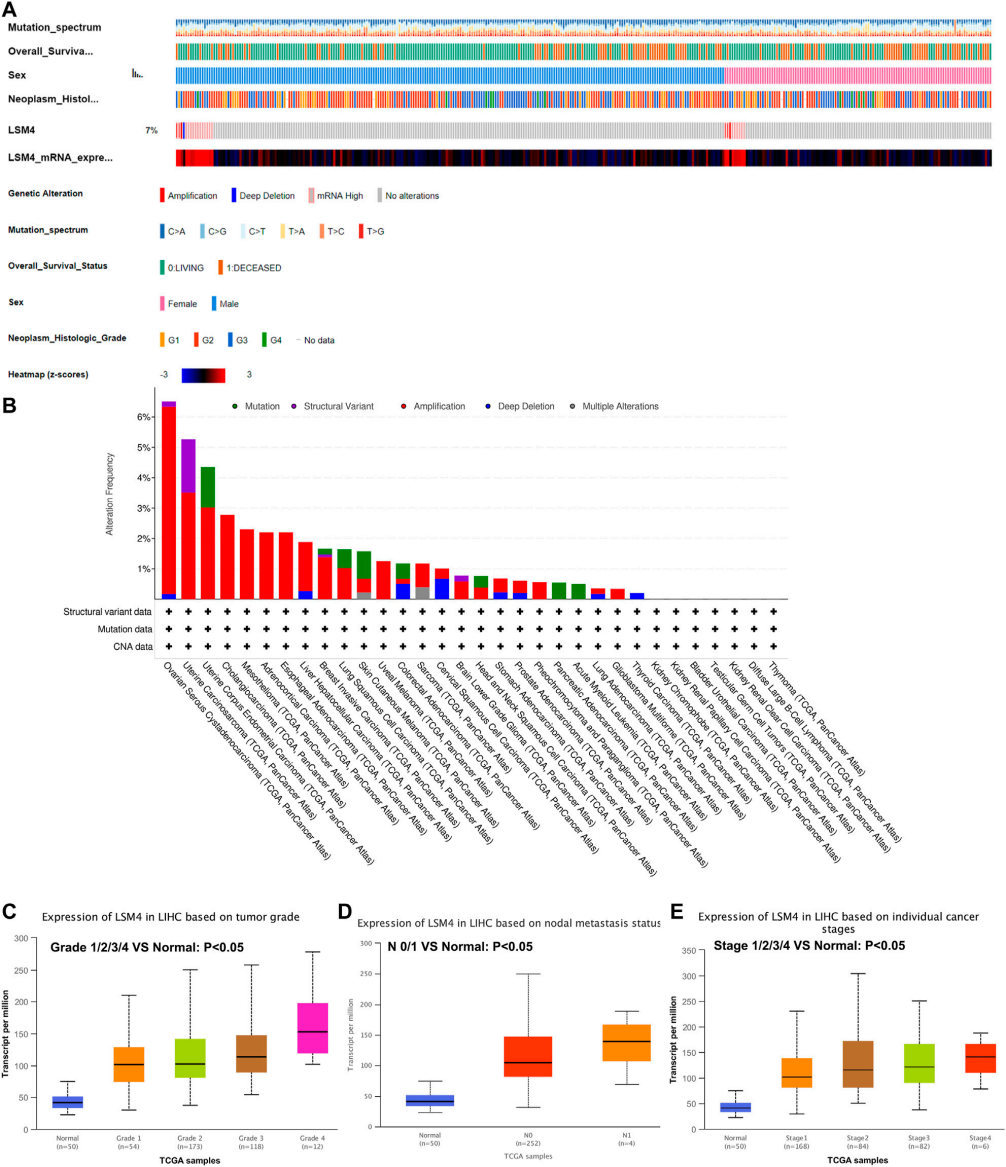
*LSM4* genetic alteration status based on cBioPortal and the relationship between *LSM4* expression and clinical characteristics of hepatocellular carcinoma based on UALCAN databases. *LSM4* was altered in 26 (7%) of the queried 372 hepatocellular carcinoma patients **(A–B)**. The expression of *LSM4* in hepatocellular carcinoma based on tumor grade, and node metastasis status, and individual cancer stages **(C–E)**.

The liver hepatocellular carcinoma (TCGA, PanCancer Atlas) was applied to analyze the genetic alterations *via* the cBioPortal database. The results revealed that the genetic alterations of *LSM4* were detected in 26 (7%) of the 372 patients. The genetic alterations of *LSM4* were mainly deep deletion, high mRNA expression, and amplification (Figure 3A). Our further study found that amplification was the most major mutation type (Figure 3B).

### Relationship Between *LSM4* Expression and the Clinical Characteristics

The relationship between *LSM4* expression and the clinical characteristics was analyzed based on the UALCAN database. As shown in Figures 3C–E, we explored the expression of *LSM4* based on cancer stage and found that *LSM4* expression was higher in advanced clinical stages (Figure 3E). After analyzing the expression of *LSM4* based on lymph node metastasis status and tumor grade, the same upward trend was also observed in our study (Figures 3C,D). In summary, our study shows that *LSM4* expression is related to cancer stage, tumor grade, and lymph node metastasis status.

### Promising Diagnostic Value of *LSM4* Overexpression in HCC

**FIGURE 4:**
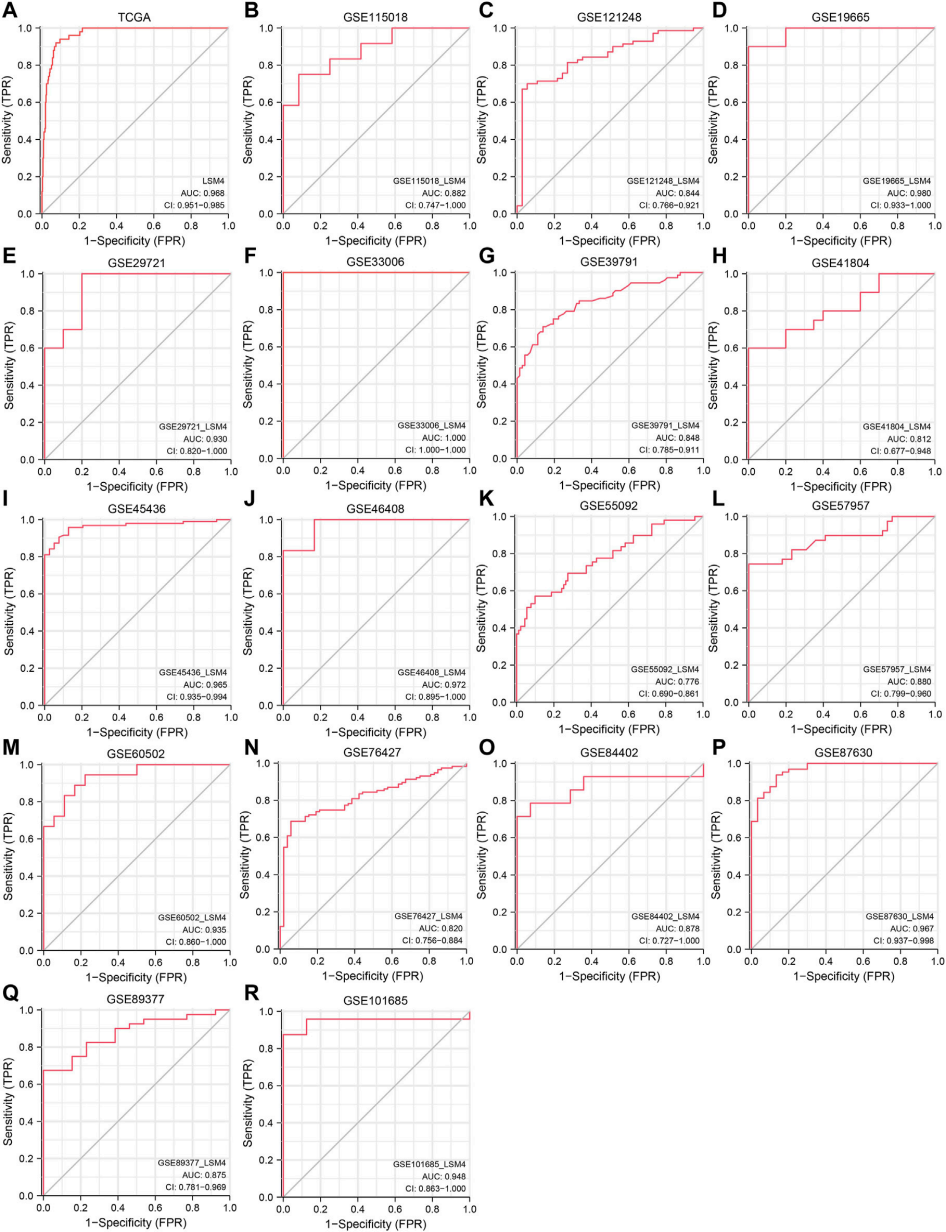
Diagnosis value of *LSM4*. The receiver operating characteristic (ROC) curve of non-HCC tissues and HCC tissues. tissues.**(A)** TCGA. **(B)** GSE115018. **(C)** GSE121248. **(D)** GSE19665. **(E)** GSE29721. **(F)** GSE33006. **(G)** GSE39791. **(H)** GSE41804. **(I)** GSE45436. **(J)** GSE46408. **(K)** GSE55092. **(L)** GSE57957. **(M)** GSE60502. **(N)** GSE76427. **(O)** GSE84402. **(P)** GSE87630. **(Q)** GSE8937. **(R)** GSE101685.

**FIGURE 5:**
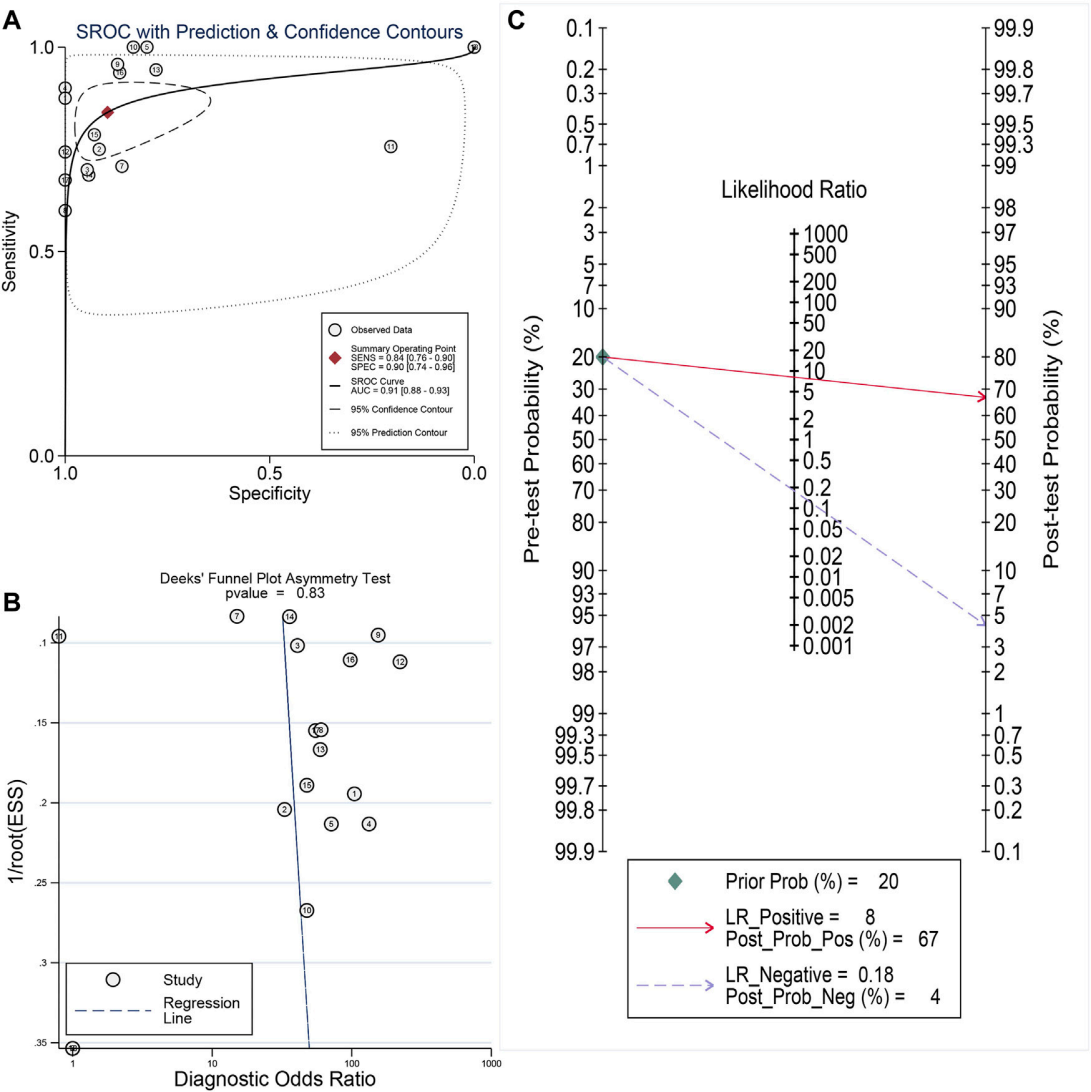
Comprehensive analysis of the diagnostic significance of *LSM4* in hepatocellular carcinoma. The summary receiver operating characteristic (sROC) curve exhibited a tremendous distinguish capacity of *LSM4* in HCC and non-HCC tissues **(A)**. Deeks’ Funnel Plot, *p*-value >0.50, indicating no publication bias **(B)**. Fagan’s plot also addressed the diagnostic value of *LSM4* for HCC patients **(C)**.

**FIGURE 6:**
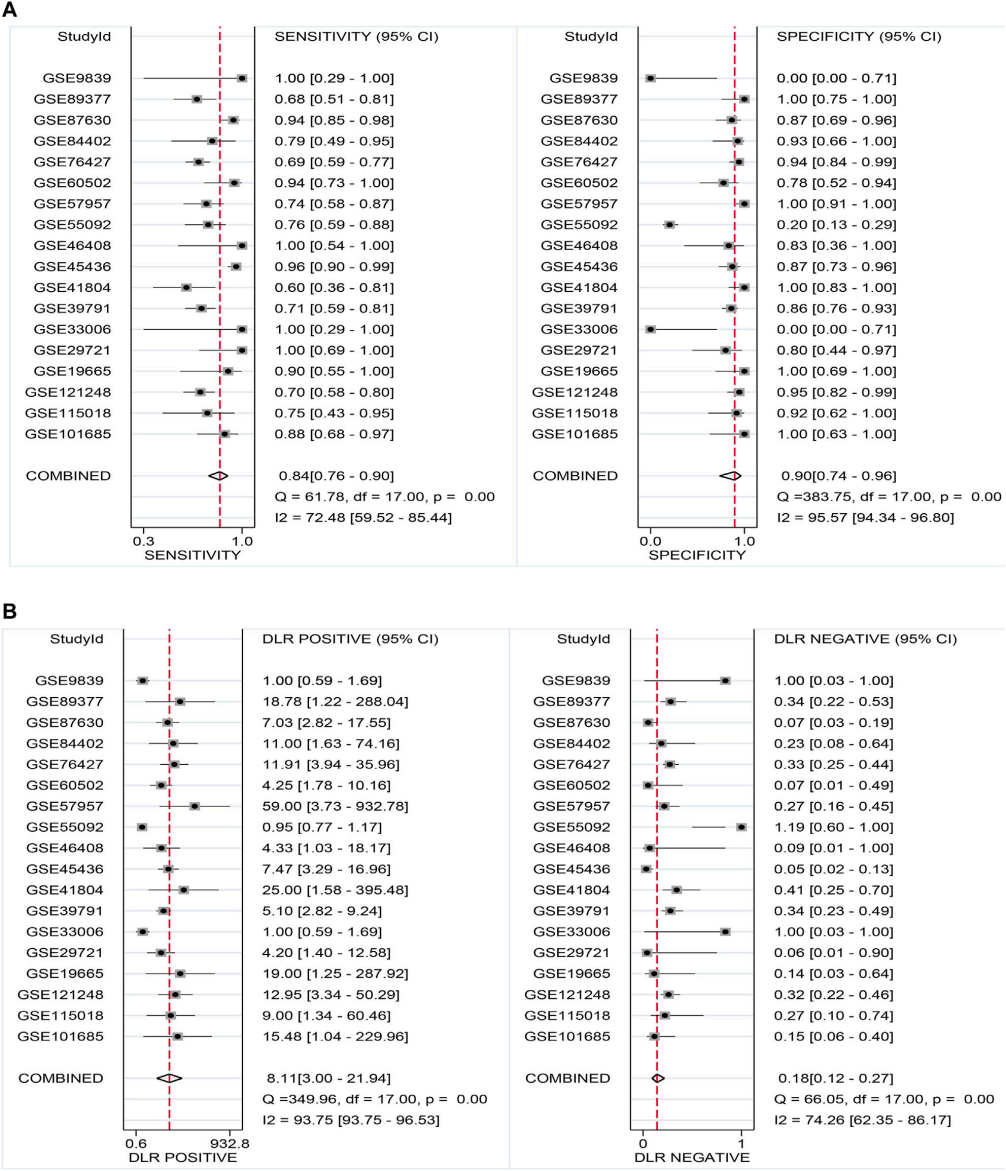
Sensitivity and specificity as well as DLR positive and DLR negative. **(A)** Sensitivity and specificity values of included studies. **(B)** DLR positive and DLR negative of included studies.

Current research results showed that *LSM4* had a significant distinguish ability for HCC and the corresponding non-HCC tissues (All AUC> 0.77; Figure 4A–R). Regarding the sROC curve analysis, the AUC, sensitivity, and specificity were 0.91 (95% CI: 0.88–0.93), 0.84 (95% CI: 0.76–0.90), and 0.90 (95% CI: 0.74–0.96), respectively (Figures 5A, 6A). Besides, the positive diagnostic likelihood ratio (DLRP) of 8.11 (95% CI: 3.00–21.94) and negative diagnostic likelihood ratio (DLRN) of 0.18 (95% CI: 0.12–0.27) were observed (Figure 6B). According to the Deeks’ funnel test, no publication bias was detected in our study (*p* = 0.83, Figure 5B). In the Fagan chart, when the pre-test probability was 20%, the post-test probability of HCC using *LSM4* for a positive test was 67%, and the probability of a negative test was 4%, indicating that *LSM4* had the potential to be an effective biomarker for HCC (Figure 5C). The above results showed that *LSM4* had excellent discrimination ability and had promising diagnostic value for HCC.

### *LSM4* Overexpression Predicted Poor Survival

**FIGURE 7:**
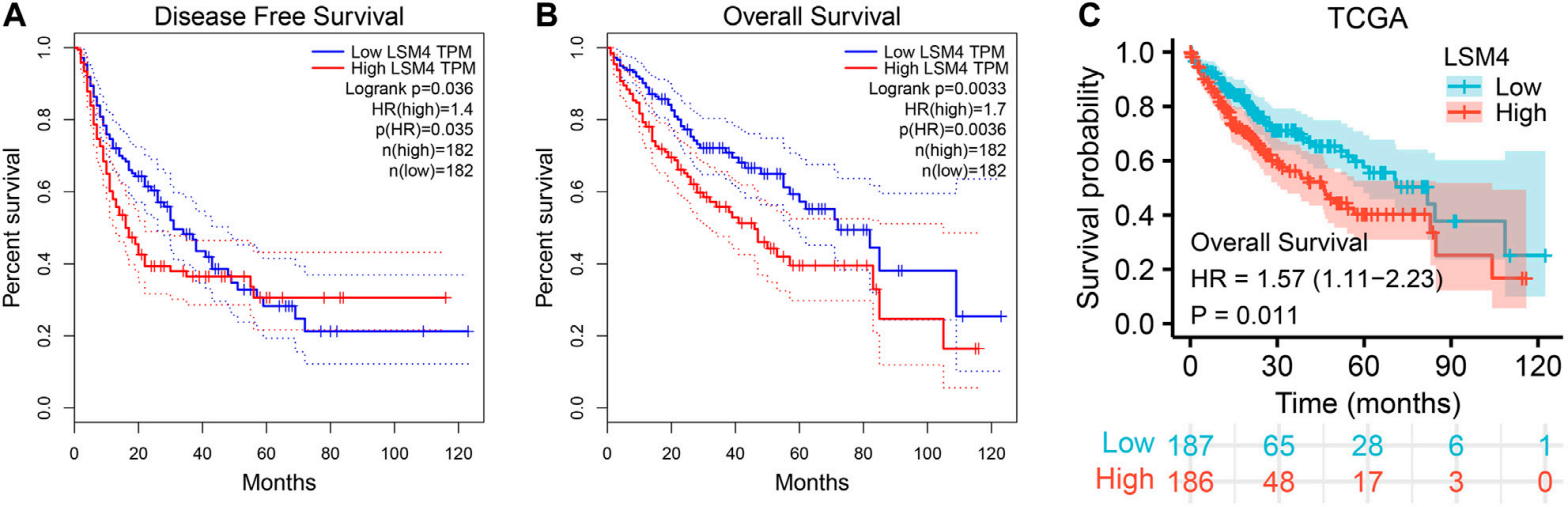
Survival curves of overall survival (OS) and disease-free survival (DFS) in hepatocellular carcinoma. Kaplan–Meier curves of hepatocellular carcinoma patients OS and DFS in patients with different *LSM4* expression based on GEPIA database **(A–B)**. Kaplan–Meier curves of OS grouped by *LSM4* expression in hepatocellular carcinoma validated by TCGA datasets **(C)**.

To determine the prognostic significance of *LSM4* expression in patients with HCC, we used the GEPIA database to detect the correlation of DFS, OS and *LSM4* expression. We observed that *LSM4* overexpression was significantly associated with shorter DFS (HR = 1.4, *p* = 0.035, Figure 7A) and OS (HR = 1.7, *p* = 0.0036, Figure 7B). In addition, based on the TCGA dataset, we divided patients into two groups (high and low groups) based on the median expression level of *LSM4* and then constructed a Kaplan-Meier chart for survival analysis. We also observed a significant association between the high expression of *LSM4* and poor OS (HR = 1.57, *p* = 0.011, Figure 7C). Furthermore, univariate and multivariate Cox regression analyses were performed on *LSM4* expression (high and low expression were divided according to the median of *LSM4* expression value) and other clinicopathological characteristics (such as AFP level, age, gender, stage, histologic grade, Fibrosis ishak score). Interestingly, the result of high *LSM4* expression was associated with poor OS was observed in both univariate and multivariate Cox regression analysis (Table 2). This suggested that overexpressed *LSM4* was independently associated with poor OS of patients with HCC.

**TABLE 2:** Univariate and multivariate cox analysis of hepatocellular carcinoma patients’ overall survival.

Characteristics	Total(N)	Univariate analysis		Multivariate analysis	
		Hazard ratio (95% CI)	*p* Value	Hazard ratio (95% CI)	*p* Value
T stage	370	—	—	—	—
T1	183	Reference	—	—	—
T2	94	1.431 (0.902–2.268)	0.128	0.000 (0.000-Inf)	0.995
T3	80	2.674 (1.761–4.060)	<0.001	1.010 (0.136–7.522)	0.992
T4	13	5.386 (2.690–10.784)	<0.001	2.484 (0.280–22.041)	0.414
Pathologic stage	349	—	—	—	—
Stage I	173	Reference	—	—	—
Stage II	86	1.417 (0.868–2.312)	0.164	2,710,301.305 (0.000-Inf)	0.995
Stage III	85	2.734 (1.792–4.172)	<0.001	2.626 (0.345–19.978)	0.351
Stage IV	5	5.597 (1.726–18.148)	0.004	1.557 (0.111–21.937)	0.743
Tumor status	354	—	—	—	—
Tumor free	202	Reference	—	—	—
With tumor	152	2.317 (1.590–3.376)	<0.001	1.719 (1.069–2.764)	0.025
Gender	373	—	—	—	—
** **Female	121	Reference	—	—	—
** **Male	252	0.793 (0.557–1.130)	0.200		
Race	361	—	—	—	—
** **Asian	159	Reference	—	—	—
** **Black or African American	17	1.585 (0.675–3.725)	0.290	—	—
** **White	185	1.323 (0.909–1.928)	0.144	—	—
Age	373	—	—	—	—
** **≤60	177	Reference	—	—	—
** **>60	196	1.205 (0.850–1.708)	0.295	—	—
BMI	336	—		—	—
** **≤25	177	Reference		—	—
** **>25	159	0.798 (0.550–1.158)	0.235	—	—
Histologic grade	368	—	—	—	—
** **G1	55	Reference		—	—
** **G2	178	1.162 (0.686–1.969)	0.576	—	—
** **G3	123	1.185 (0.683–2.057)	0.545	—	—
** **G4	12	1.681 (0.621–4.549)	0.307	—	—
AFP level (ng/ml)	279	—	—	—	—
** **≤400	215	Reference	—	—	—
** **>400	64	1.075 (0.658–1.759)	0.772	—	—
Fibrosis ishak score	214	—	—	—	—
** **0	75	Reference	—	—	—
** **1/2	31	0.935 (0.437–2.002)	0.864	—	—
** **3/4	28	0.698 (0.288–1.695)	0.428	—	—
** **5/6	80	0.737 (0.410–1.325)	0.308	—	—
N stage	258	—	—	—	—
** **N0	254	Reference	—	—	—
** **N1	4	2.029 (0.497–8.281)	0.324	—	—
M stage	272	—	—	—	—
** **M0	268	Reference	—	—	—
** **M1	4	4.077 (1.281–12.973)	0.017	—	—
LSM4	373	—	—	—	—
Low	187	Reference	—	—	—
High	186	1.619 (1.144–2.292)	0.007	1.692 (1.062–2.694)	0.027

### Identification of CEGs and Their Clinical Value in HCC

**FIGURE 8:**
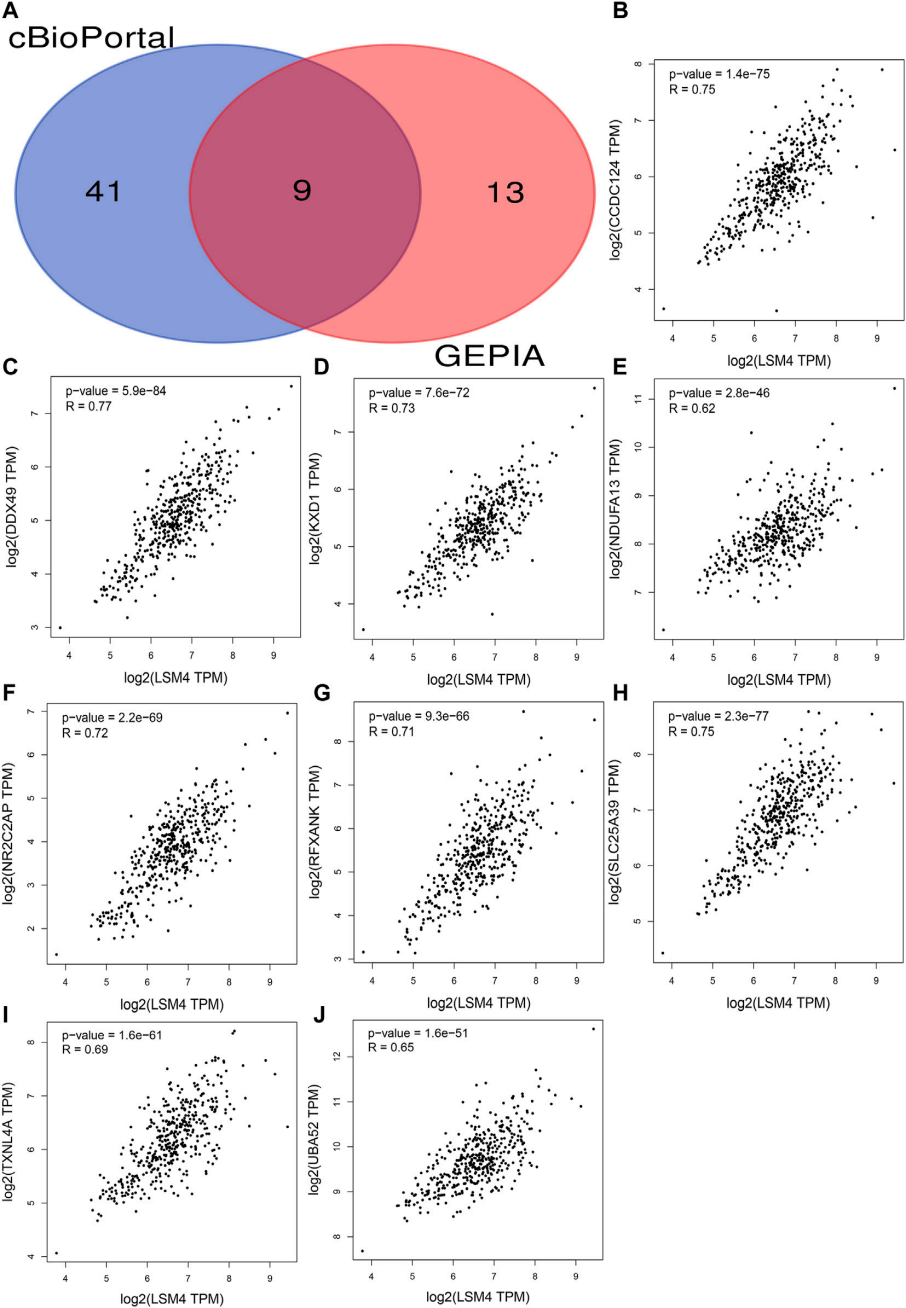
Identification co-expressed genes (CEGs) of *LSM4* in hepatocellular carcinoma. Venn diagram for the nine intersection genes of *LSM4* positively related CEGs identified by the cBioPortal and the GEPIA databases **(A)**. The scatter plot shows the significant positive correlation between *LSM4* and nine CEGs **(B–J)**.

**FIGURE 9:**
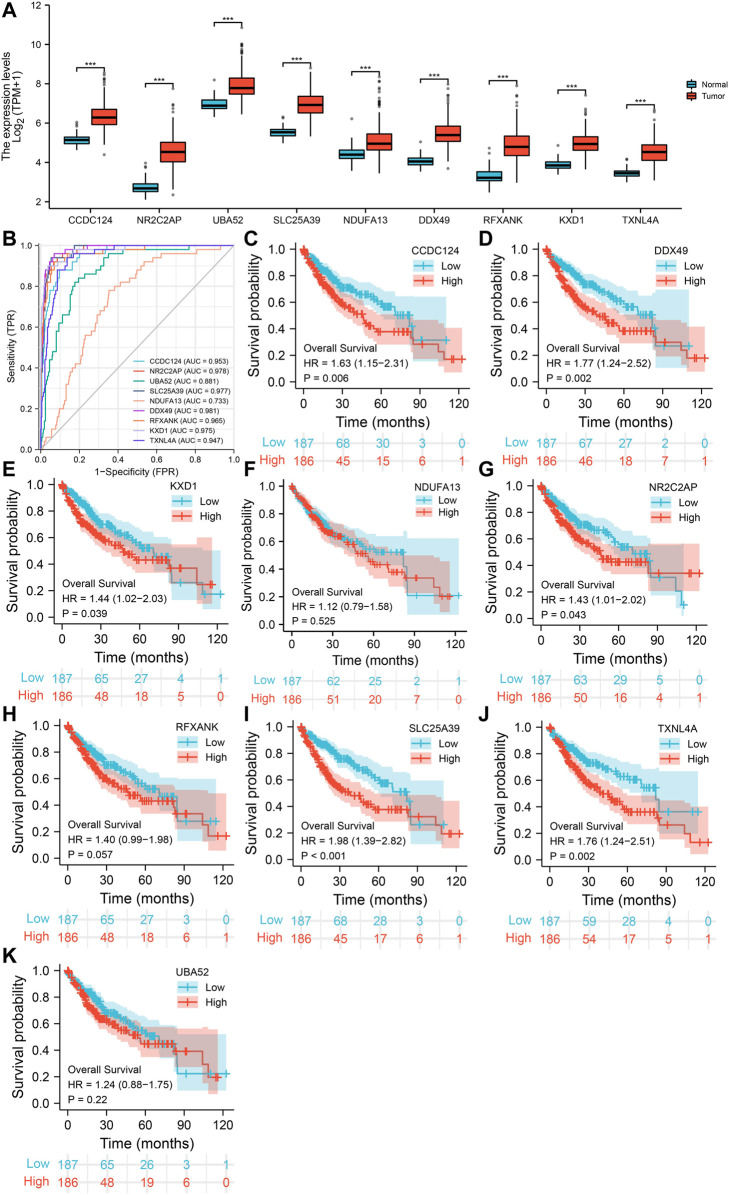
The expression of nine co-expressed genes (CEGs) in hepatocellular carcinoma and their diagnostic and prognostic significance based on TCGA dataset. The elevated expression of nine CEGs in HCC tissues **(A)**. The receiver operating characteristic (ROC) curve of *LSM4* in non-HCC tissues and HCC tissues **(B)**. Kaplan–Meier curves of overall survival (OS) grouped by *LSM4* expression in hepatocellular carcinoma **(C–K)**.

The correlation coefficients of *LSM4* and other genes were obtained through the GEPIA and cBioPortal databases. Subsequently, CEGs meeting the criteria of correlation coefficient ≥0.60 with *p*-value <0.05 were obtained for further analysis. In the GEPIA and cBioPortal databases, 22 and 50 *LSM4* positively correlated CEGs were identified, respectively, and a total of nine crossing CEGs were obtained (Figure 8A). The scatter plot shows the significant positive correlation between *LSM4* and nine CEGs (Figures 8B–J). In addition, our research based on the TCGA dataset found that the expression level of nine CEGs was significantly up-regulated in HCC tissues (Figure 9A). Next, we also explored the clinical significance of nine CEGs expressions in HCC. Our results show that the above genes had excellent recognition ability for HCC tissues and non-HCC tissues, showing the promising diagnostic value (2 CEGs AUC>0.7, seven CEGs AUC>0.90, Figure 9B). Subsequent survival analysis also showed that six CEGs were closely associated with the poor OS of patients with HCC, while the other three CEGs showed no significant relation to the OS of HCC patients (Figures 9C–K). In summary, six CEGs, including CCDC124, NR2C2AP, SLC25A39, DDX49, KXD1, TXNL4A, showed promising diagnostic and prognostic significance (AUC>0.90; HR > 1.40, *p* < 0.05), which may indicate showing its attractive research value in HCC.

### *LSM4*-Associated Signal Pathways in HCC

**FIGURE 10:**
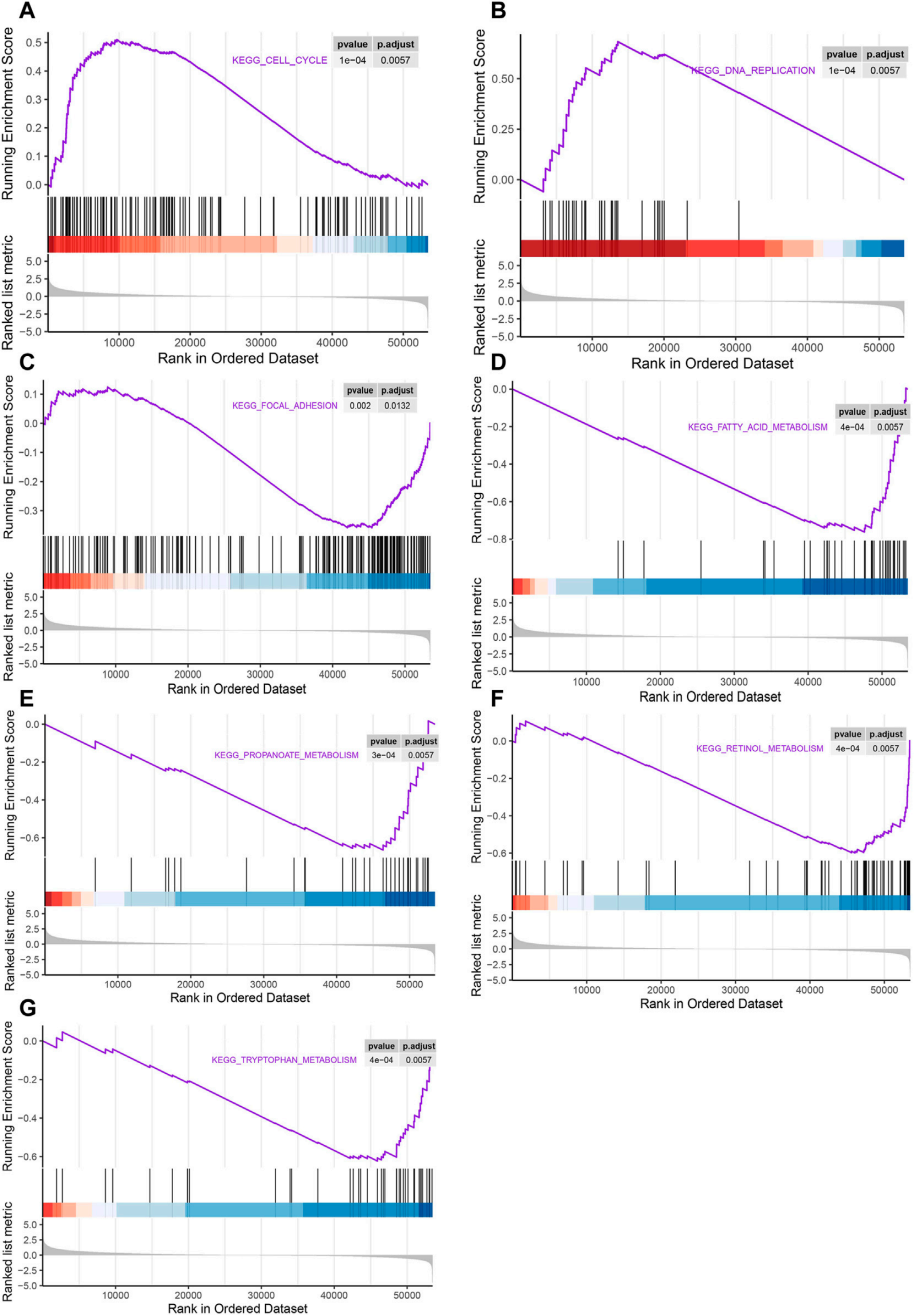
Gene set enrichment analysis (GSEA) of pathways associated with *LSM4* expression. The LSM4 expression was significantly correlated with the cell cycle, DNA replication, focal adhesion and multiple metabolism-associated pathways **(A–G)**.

In order to determine the cancer-related signaling pathways related to *LSM4*, GSEA analysis was performed using the *LSM4* expression data contained in the TCGA dataset. The results showed that the cell cycle and DNA replication pathways were significantly activated in patients with *LSM4* overexpression (Figures 10A,B). On the contrary, the *LSM4* overexpression was negatively correlated with the focal adhesion pathway (Figure 10C). Besides, *LSM4* overexpression was also significantly negatively correlated with multiple metabolism-associated pathways, such as fatty acid metabolism, propanoate acid metabolism, retinol metabolism, and tryptophan metabolism (Figures 10D–G).

## Discussion

As a highly malignant tumor with a poor prognosis, hepatocellular carcinoma (HCC) seriously threatens human health and causes a great economic burden to patients and society (Chen et al., 2016; Yan and Fan, 2019). Although plenty of studies in recent years have reported a variety of biomarkers for HCC, the currently available are still insufficient (Pang et al., 2020; Xing et al., 2020; Cai et al., 2021; Dolicka et al., 2021). Therefore, it is necessary to identify novel biomarkers for early diagnosis and prognostic monitoring of HCC. The main purpose of our current study is to identify the clinical significance of *LSM4* in HCC and try to reveal its underlying molecular mechanism using bioinformatics methods.

First, the expression pattern and clinical significance of *LSM4* in HCC were explored using the GEO and TCGA datasets. In our study, the combined SMD reached 1.56 (95% CI: 1.29–1.84, *p* < 0.001), and there was no significant publication bias observed in our study, suggesting a significant overexpression of *LSM4* in HCC. Through ROC curve analysis, the excellent performance of *LSM4* to distinguish HCC tissues from non-HCC liver tissues was observed (All AUC> 0.77). Subsequent sROC analysis also confirmed the above conclusion (AUC = 0.91, 95% CI: 0.88–0.93). In addition, its higher expression also appeared to be associated with higher tumor grade, lymph node metastasis status, pathological stage and poor patient prognosis. In addition, univariate and multivariate Cox regression analysis, including the main clinical features, showed that *LSM4* expression was independently associated with the poor OS in patients with HCC (both HR > 1.6, *p* < 0.05). Summarily, the current evidence indicated that *LSM4* might act as a cancer-promoting factor and participate in the occurrence and malignant progression of HCC, and it has the potential as the diagnostic biomarker of HCC and the predictor of poor prognosis.

Subsequently, we identified nine CEGs positively correlated with *LSM4* in HCC to further analyze the mechanisms. Our study showed that all CEGs were significantly up-regulated in HCC. Further study found that six CEGs, including CCDC124, NR2C2AP, SLC25A39, DDX49, KXD1, TXNL4A, had good diagnostic value and were related to poor OS in HCC patients (all AUC>0.90; all HR > 1.40, *p* < 0.05). According to the previous reports, CCDC124 was significantly overexpressed in endometrial cancer (EC), hepatocellular carcinoma (HCC), colorectal cancer (CRC), and ovarian cancer (OC) was related to tumor cell division, tumor heterogeneity and drug resistance (Lu et al., 2020; Arslan et al., 2021). The expression of DDX49 was significantly up-regulated in HCC, and knocking it down could suppress the growth and metastasis of HCC tumors, while overexpression of DDX49 could enhance the above procession (Dai et al., 2021). In addition, DDX49 participated in forming the SNHG20/miR-342/DDX49 axis, thereby participating in the positive regulation of lung adenocarcinoma cell proliferation, invasion and apoptosis (Wang et al., 2020). Another study proved that DDX49 participated in the promotion of the growth of NSCLC cells and lymph node metastasis *via* increasing the Akt/β-catenin pathway (Lian et al., 2020). In NSCLC, KXD1 overexpression was positively correlated with cancer invasion and metastasis and negatively correlated with non-surgical anti-cancer treatment resistance (Wang et al., 2021). NR2C2AP, which was highly expressed in NSCLC, also known as TRA16, could promote cancer cell growth by enhancing the ERβ signaling pathway and was associated with lymph node metastasis and poor OS (Fang et al., 2013). Since that TXNL4A and SLC25A39 were rarely reported in cancer, we did not discuss them in-depth. Although not all six CEGs have been reported in HCC, combined with our study and previous reports, the above genes seemed closely related to tumor growth and metastasis. Given the significant positive correlation between *LSM4* and the above CEGs in HCC, we speculate that *LSM4* may cooperate with the six positively correlated CEGs in the promotion of the growth and metastasis of HCC cells.

The results from the GSEA analysis showed that the *LSM4* overexpression in HCC was strongly associated with the activation of cell proliferation-related pathways, including DNA replication and cell cycle pathways. Cell cycle disorder was one of the hallmarks of cancer (Stewart et al., 2003). Previous studies demonstrated that cell cycle disorders could lead to uncontrolled cell proliferation, leading to cancer development (Yang et al., 2017; Zhou et al., 2018). Disorders of cell proliferation and metastasis were important causes of cancer development (Yang et al., 2019). In addition, our study also showed that there was a significant correlation between the focal adhesion pathway and *LSM4* overexpression. Numerous studies have reported that the focal adhesion pathway had a crucial effect on cancer metastasis and invasive behavior. Among the genes represented in this pathway was FAK, which may have an essential role in promoting tumorigenesis and metastasis (Luo and Guan, 2010; Ocak et al., 2010; Zhang et al., 2021). Another study showed that activation of focal adhesion pathway mediated the survival, invasion, proliferation and drug resistance of HCC cells. Besides, the results from GSEA analysis also indicated that *LSM4* overexpression was related to multiple metabolic pathways, such as fatty acid metabolism, propionic acid metabolism, retinol metabolism, and tryptophan metabolism. In recent years, tumor metabolism has gradually become one of the research hotspots (Liu C et al., 2021). It played a key role in the malignant biological behavior of cancer and was beneficial to the survival, proliferation, invasion and metastasis of cancer cells (Ma and Zong, 2020). For example, fatty acid metabolism played an essential role in the progression and metastasis of various cancers, including PC and HCC (Sunami et al., 2021). In general, the results of GSEA analysis supported the previous speculation that overexpression of *LSM4* seemed to be more closely related to the promotion of growth and metastasis of HCC cells. Besides, it may be through the regulation of crucial pathways, such as cell cycle, focal adhesion and metabolism-related pathways, to play the above role.

This study has some obvious advantages. Based on the integrated 18 datasets from the TCGA and GEO databases, we comprehensively identified the overexpression of *LSM4* in HCC and its promising diagnostic and prognostic value. Datasets from multiple sources and large sample size ensured the credibility of our research. However, the limitations of this study also could not be ignored. First, although the random-effects model was applied, the influence of higher heterogeneity research could not be completely eliminated; secondly, our study was limited *in vitro* studies, and more *in vivo* and vitro studies were still needed in the future to demonstrate our conclusions and clarify the specific mechanism of *LSM4* in the growth and metastasis of HCC.

## Conclusions

In conclusion, overexpressed *LSM4* may serve as a promising diagnostic and prognostic biomarker for HCC. Besides, *LSM4* may play a synergistic effect with CEGs in promoting the growth and metastasis of HCC cells *via* regulating crucial pathways such as cell cycle, focal adhesion, and metabolism-associated pathways.

## Abbreviations

AFP, alpha-fetoprotein; BRCA, breast cancer; CEGs, co-expressed genes; CRC, colorectal cancer; DFS, Disease-Free Survival; DLRN, negative diagnostic likelihood ratio; EC, endometrial cancer; FDR, false discovery rate; GEO, Gene Expression Omnibus; GSEA, Gene set enrichment analysis; HCC, Hepatocellular carcinoma; LSM, Smith-like; LUSC, lung squamous cell carcinoma; OS, overall survival; OC, ovarian cancer; PC, pancreatic cancer; ROC, receiver operating characteristic; SMD, standardized mean difference; sROC, summary receiver operating characteristic; TCGA, The Cancer Genome Atlas.

## Data Availability

The original contributions presented in the study are included in the article/supplementary material, further inquiries can be directed to the corresponding authors.

## References

[B1] Arslan Ö. Soylu N. K. Akillilar P. T. Tazebay U. H. (2021). Coiled-coil Domain-Containing Protein-124 (Ccdc124) Is a Novel RNA Binding Factor Up-Regulated in Endometrial, Ovarian, and Urinary Bladder Cancers. Cancer Biomark. 31, 149–164. 10.3233/cbm-200802 33896821 PMC12500014

[B2] Bray F. Ferlay J. Soerjomataram I. Siegel R. L. Torre L. A. Jemal A. (2018). Global Cancer Statistics 2018: GLOBOCAN Estimates of Incidence and Mortality Worldwide for 36 Cancers in 185 Countries. CA: A Cancer J. Clin. 68, 394–424. 10.3322/caac.21492 30207593

[B3] Cai C. Zhang Y. Hu X. Hu W. Yang S. Qiu H. (2021). CDT1 Is a Novel Prognostic and Predictive Biomarkers for Hepatocellular Carcinoma. Front. Oncol. 11, 721644. 10.3389/fonc.2021.721644 34631549 PMC8497762

[B4] Chen M. Luo F. Yu J. Xiang G. Jiang D. Pu X. (2016). Common Functional Polymorphism within miR-146a and miR-196a-2 as Susceptibility Loci for Hepatocellular Carcinoma: An Updated Meta-Analysis. Meta Gene. 7, 40–47. 10.1016/j.mgene.2015.11.002 26862480 PMC4707244

[B5] Dai H. Feng J. Nan Z. Wei L. Lin F. Jin R. (2021). Morphine May Act via DDX49 to Inhibit Hepatocellular Carcinoma Cell Growth. Aging 13, 12766–12779. 10.18632/aging.202946 33952717 PMC8148497

[B6] Dolicka D. Sobolewski C. Gjorgjieva M. Correia de Sousa M. Berthou F. De Vito C. (2021). Tristetraprolin Promotes Hepatic Inflammation and Tumor Initiation but Restrains Cancer Progression to Malignancy. Cell Mol. Gastroenterol. Hepatol. 11, 597–621. 10.1016/j.jcmgh.2020.09.012 32987153 PMC7806869

[B7] Fang F. Zheng Q. Zhang J. Dong B. Zhu S. Huang X. (2013). Testicular Orphan Nuclear Receptor 4-associated Protein 16 Promotes Non-small Cell Lung Carcinoma by Activating Estrogen Receptor β and Blocking Testicular Orphan Nuclear Receptor 2. Oncol. Rep. 29, 297–305. 10.3892/or.2012.2107 23129017 PMC3583481

[B8] Hou W. Zhang Y. (2021). Circ_0025033 Promotes the Progression of Ovarian Cancer by Activating the Expression of LSM4 via Targeting miR-184. Pathol. Res. Pract. 217, 153275. 10.1016/j.prp.2020.153275 33285422

[B9] Li W. Li X. Gao L.-N. You C.-G. (2020). Integrated Analysis of the Functions and Prognostic Values of RNA Binding Proteins in Lung Squamous Cell Carcinoma. Front. Genet. 11, 185. 10.3389/fgene.2020.00185 32194639 PMC7066120

[B10] Lian X. Xiang D. Peng C. Chen J. Liao M. Sun G. (2020). DDX49 Is a Novel Biomarker and Therapeutic Target for Lung Cancer Metastases. J. Cel Mol. Med. 24, 1141–1145. 10.1111/jcmm.14734 PMC6933356 31749282

[B11] Liu C C. Jin Y. Fan Z. (2021). The Mechanism of Warburg Effect-Induced Chemoresistance in Cancer. Front. Oncol. 11, 698023. 10.3389/fonc.2021.698023 34540667 PMC8446599

[B12] Liu Y Y. Veeraraghavan V. Pinkerton M. Fu J. Douglas M. W. George J. (2021). Viral Biomarkers for Hepatitis B Virus-Related Hepatocellular Carcinoma Occurrence and Recurrence. Front. Microbiol. 12, 665201. 10.3389/fmicb.2021.665201 34194408 PMC8236856

[B13] Lu W. Fu D. Kong X. Huang Z. Hwang M. Zhu Y. (2020). FOLFOX Treatment Response Prediction in Metastatic or Recurrent Colorectal Cancer Patients via Machine Learning Algorithms. Cancer Med. 9, 1419–1429. 10.1002/cam4.2786 31893575 PMC7013065

[B14] Luo M. Guan J.-L. (2010). Focal Adhesion Kinase: a Prominent Determinant in Breast Cancer Initiation, Progression and Metastasis. Cancer Lett. 289, 127–139. 10.1016/j.canlet.2009.07.005 19643531 PMC2854647

[B15] Ma L. Zong X. (2020). Metabolic Symbiosis in Chemoresistance: Refocusing the Role of Aerobic Glycolysis. Front. Oncol. 10, 5. 10.3389/fonc.2020.00005 32038983 PMC6992567

[B16] Nordenstedt H. White D. L. El-Serag H. B. (2010). The Changing Pattern of Epidemiology in Hepatocellular Carcinoma. Dig. Liver Dis. 42, S206–S214. 10.1016/s1590-8658(10)60507-5 20547305 PMC3392755

[B17] Ocak S. Yamashita H. Udyavar A. R. Miller A. N. Gonzalez A. L. Zou Y. (2010). DNA Copy Number Aberrations in Small-Cell Lung Cancer Reveal Activation of the Focal Adhesion Pathway. Oncogene 29, 6331–6342. 10.1038/onc.2010.362 20802517 PMC4637980

[B18] Pang Y. Y. Li J. D. Gao L. Yang X. Dang Y. W. Lai Z. F. (2020). The Clinical Value and Potential Molecular Mechanism of the Downregulation of MAOA in Hepatocellular Carcinoma Tissues. Cancer Med. 9, 8004–8019. 10.1002/cam4.3434 32931665 PMC7643659

[B19] Stewart Z. A. Westfall M. D. Pietenpol J. A. (2003). Cell-cycle Dysregulation and Anticancer Therapy. Trends Pharmacol. Sci. 24, 139–145. 10.1016/s0165-6147(03)00026-9 12628359

[B20] Streicher K. L. Yang Z. Q. Draghici S. Ethier S. P. (2007). Transforming Function of the LSM1 Oncogene in Human Breast Cancers with the 8p11-12 Amplicon. Oncogene 26, 2104–2114. 10.1038/sj.onc.1210002 17001308 PMC2435249

[B21] Sunami Y. Rebelo A. Kleeff J. (2021). Lipid Droplet-Associated Factors, PNPLA3, TM6SF2, and HSD17B Proteins in Hepatopancreatobiliary Cancer. Cancers (Basel) 13 (17), 4391. 10.3390/cancers13174391 34503201 PMC8431307

[B22] Ta H. D. K. Wang W. J. Phan N. N. An Ton N. T. Anuraga G. Ku S. C. (2021). Potential Therapeutic and Prognostic Values of LSM Family Genes in Breast Cancer. Cancers (Basel) 13 (19), 4902. 10.3390/cancers13194902 34638387 PMC8508234

[B23] Tang W. Li J. Chang X. Jia L. Tang Q. Wang Y. (2020). Construction of a Novel Prognostic-Predicting Model Correlated to Ovarian Cancer. Biosci. Rep. 40 (8), BSR20201261. 10.1042/BSR20201261 32716025 PMC7414523

[B24] Wang W. Wei C. (2020). Advances in the Early Diagnosis of Hepatocellular Carcinoma. Genes Dis. 7, 308–319. 10.1016/j.gendis.2020.01.014 32884985 PMC7452544

[B25] Wang X. Gu G. Zhu H. Lu S. Abuduwaili K. Liu C. (2020). LncRNA SNHG20 Promoted Proliferation, Invasion and Inhibited Cell Apoptosis of Lung Adenocarcinoma via Sponging miR ‐342 and Upregulating DDX49. Thorac. Cancer 11, 3510–3520. 10.1111/1759-7714.13693 33089952 PMC7705913

[B26] Wang S. Ma H. Li H. Liu Q. Huang S. Huang L. (2021). Alternatively Expressed Transcripts Analysis of Non-small Cell Lung Cancer Cells under Different Hypoxic Microenvironment. J. Oncol. 2021, 5558304. 10.1155/2021/5558304 33936200 PMC8055392

[B27] Watson P. M. Miller S. W. Fraig M. Cole D. J. Watson D. K. Boylan A. M. (2008). CaSm (LSm-1) Overexpression in Lung Cancer and Mesothelioma Is Required for Transformed Phenotypes. Am. J. Respir. Cel Mol. Biol. 38, 671–678. 10.1165/rcmb.2007-0205oc PMC2396246 18218995

[B28] Xie Z. Zhou F. Yang Y. Li L. Lei Y. Lin X. (2018). Lnc-PCDH9-13:1 Is a Hypersensitive and Specific Biomarker for Early Hepatocellular Carcinoma. EBioMedicine 33, 57–67. 10.1016/j.ebiom.2018.06.026 30045829 PMC6085584

[B29] Xing J. Tian Y. Ji W. Wang X. (2020). Comprehensive Evaluation of SPATS2 Expression and its Prognostic Potential in Liver Cancer. Medicine 99, e19230. 10.1097/md.0000000000019230 32118724 PMC7478581

[B30] Xue R. Hua L. Xu W. Gao Y. Pang Y. Hao J. (2018). Derivation and Validation of the Potential Core Genes in Pancreatic Cancer for Tumor-Stroma Crosstalk. Biomed. Res. Int. 2018, 4283673. 10.1155/2018/4283673 30519576 PMC6241336

[B31] Yan S. Y. Fan J. G. (2019). Advances in the Study of Non-infectious Liver Diseases and Precancerous Lesions of Hepatocellular Carcinoma. Zhonghua Gan Zang Bing Za Zhi 27, 487–490. 10.3760/cma.j.issn.1007-3418.2019.07.003 31357772 PMC12769220

[B32] Yang C.-Y. Hsieh C.-C. Lin C.-K. Lin C.-S. Peng B. Lin G.-J. (2017). Danshen Extract Circumvents Drug Resistance and Represses Cell Growth in Human Oral Cancer Cells. BMC Complement. Altern. Med. 17, 555. 10.1186/s12906-017-2063-y 29284481 PMC5747158

[B33] Yang Y. Chen D. Liu H. Yang K. (2019). Increased Expression of lncRNA CASC9 Promotes Tumor Progression by Suppressing Autophagy-Mediated Cell Apoptosis via the AKT/mTOR Pathway in Oral Squamous Cell Carcinoma. Cell Death Dis. 10, 41. 10.1038/s41419-018-1280-8 30674868 PMC6381212

[B34] Yin J. Lin C. Jiang M. Tang X. Xie D. Chen J. (2021). CENPL, ISG20L2, LSM4, MRPL3 Are Four Novel Hub Genes and May Serve as Diagnostic and Prognostic Markers in Breast Cancer. Sci. Rep. 11, 15610. 10.1038/s41598-021-95068-6 34341433 PMC8328991

[B35] Zhang Z. Yu Y. Zhang P. Ma G. Zhang M. Liang Y. (2021). Identification of NTRK3 as a Potential Prognostic Biomarker Associated with Tumor Mutation burden and Immune Infiltration in Bladder Cancer. BMC Cancer 21, 458. 10.1186/s12885-021-08229-1 33894748 PMC8070296

[B36] Zhou H. Bao J. Zhu X. Dai G. Jiang X. Jiao X. (2018). Retinoblastoma Binding Protein 5 Correlates with the Progression in Hepatocellular Carcinoma. Biomed. Res. Int. 2018, 1073432. 10.1155/2018/1073432 30533424 PMC6247687

